# Bona-fide method for the determination of short range order and transport properties in a ferro-aluminosilicate slag

**DOI:** 10.1038/srep30216

**Published:** 2016-07-26

**Authors:** Konstantinos T. Karalis, Dimitrios Dellis, Georgios S. E. Antipas, Anthimos Xenidis

**Affiliations:** 1 School of Mining Engineering and Metallurgy, National Technical University of Athens, Zografou Campus, Athens 15780, Greece; 2 Greek Research & Technology Network, Mesogeion 56, Athens 11527, Greece

## Abstract

The thermodynamics, structural and transport properties (density, melting point, heat capacity, thermal expansion coefficient, viscosity and electrical conductivity) of a ferro-aluminosilicate slag have been studied in the solid and liquid state (1273–2273 K) using molecular dynamics. The simulations were based on a Buckingham-type potential, which was extended here, to account for the presence of Cr and Cu. The potential was optimized by fitting pair distribution function partials to values determined by Reverse Monte Carlo modelling of X-ray and neutron diffraction experiments. The resulting short range order features and ring statistics were in tight agreement with experimental data and created consensus for the accurate prediction of transport properties. Accordingly, calculations yielded rational values both for the average heat capacity, equal to 1668.58 J/(kg·K), and for the viscosity, in the range of 4.09–87.64 cP. The potential was consistent in predicting accurate values for mass density (i.e. 2961.50 kg/m^3^ vs. an experimental value of 2940 kg/m^3^) and for electrical conductivity (5.3–233 S/m within a temperature range of 1273.15–2273.15 K).

Ferronickel is produced by reductive smelting (RS) of nickel laterite ores in electric submerged arc furnaces (EAF)^1^,^2^,^3^. RS involves industrial infrastructures of grand physical proportions with correspondingly high requirements for electric energy, in excess of 40 MWatts per EAF. In fact, the electric energy required for RS accounts for a substantial 30% of the total operational cost and it is principally consumed in order to initiate and maintain melting of a laterite quantity under the effect of Joule heating, the ore being periodically fed into the EAF. Continuous chemical reduction of the resulting (mixed oxide) slag via excess carbon completes the process, by causing ferronickel sedimentation into the lower EAF compartment, which is regularly vacated in order to maintain the production cycle.

The key parameters affecting EAF productivity are the density and the mesoscale transport properties of the slag, principally viscosity and electrical conductivity. High temperatures favor stirring of the slag due to density fluctuations formed within the bath (buoyancy effects) and low slag viscosity^4^, the latter feeding into the efficacy of the reduction process by promoting metal/slag reaction kinetics and by facilitating mass and heat transfer^4^. The electrical conductivity of the slag, in particular, has been the focus of a large body of research devoted to the expression of its dependence on temperature and on the concentration of phases present in the bath, via empirical relations which are typically validated over a broad spectrum of slag concentrations and temperature regimes^5^,^6^. Although such relations are adequate for a high-level approach of the transport properties, they are, nonetheless, merely abstractions of the associated phenomena. In fact, in certain instances, empirical relations utilize non-physical reasoning, as is the case of summing up the concentrations of the CaO and MgO phases in the estimation of the melting point of the slag using ternary diagrams. Most importantly, empirical relations provide limited, if any, physical insight into the dependence of transport properties on short range order (SRO). For example, empirical relations do not resolve the important effect of the different oxidation states of iron^7^ on transport phenomena. Another such instance arising from molten aluminosilicates is the increase of the self-diffusivity of oxygen atoms, which results from an increase of the fraction of penta-coordinated cations such as Si, Fe^3+^ and Al 8,9. It, thus, becomes natural to enquire whether prognosis of the transport properties may be based on knowledge of the atomic structure of the slag melt by the application of first principles^9^,^10^.

Virtually all slags of consequence to RS are semiconductive^6^ and there is ample consensus that the range of their electrical conductivities may be accurately predicted by the Nernst-Einstein relationship^10^,^11^, on the provision that ionic self-diffusivities can be estimated. It is noted that self-diffusivity refers to ionic mobility in the absence of chemical gradients and is typically expressed as the mean square displacement (MSD)^9^ of each atomic species. The numerical calculation of the coefficients of self-diffusivity is profoundly dependent upon slag kinetics, which, in turn, may be derived from the atomic structure via molecular dynamics (MD); therefore, estimation of transport properties reverts to an exercise aimed at the accurate determination of atomic structure, and, in particular, of SRO.

The slag SRO is dictated by the network forming capability of the cations, which are classified into three groups, depending on their contribution towards order formation in the melt. These groups are network formers, network modifiers and amphoteric ions. Network formers (e.g., Si^4+^ and Ge^4+^) stabilize the network and, therefore, increase viscosity^6^,^7^,^12^. Network modifiers (e.g., Na^+^, K^+^, Mg^2+^, Ca^2+^, Fe^2+^, Ba^2+^ and Sr^2+^) disrupt the network and, as a result, cause a reduction of viscosity. Amphoterics (e.g., Al^3+^, Fe^3+^, Cr^3+^, B^3+^ and Zn^2+^) can act either as network formers or modifiers^6^,^7^,^13^. Alongside the role of the cations, the oxygen species – by far the most abundant species in any slag – is instrumental to SRO as it affects melt polymerization. In fact, the viscosity of ferro-aluminosilicate melts appears to be tightly correlated to atomic structure via the degree of polymerization (higher degree with increasing silica content)^14^,^15^. The degree of melt polymerization depends on the motion of three types of oxygen states: the free O^2−^ species, the non-bridging oxygen (NBO - e.g. O^−^ bonded to one Si) and bridging oxygen atoms (BO - e.g. O^0^ bonded to two Si)^14^. In fact, the presence of excess NBOs might potentially be the cause of viscosity anomalies measured in aluminosilicate systems^16^,^17^,^18^. The number of NBOs is affected from the presence of other types of polyhedra, such as oxygen tri-clusters and/or highly coordinated Si and Al atoms^16^,^17^. Typically, network polymerization is expressed as the ratio the number of NBOs divided by the number of tetrahedrally-coordinated network-forming cations^8^. The viscosity of most molten silicates is also known to depend on the concentration of NBOs, the number of the latter reported to depend on the Al_2_O_3_/CaO ratio^13^. It is of course expected that, on a fundamental level, such a ratio of phases must precipitate from the atomic coordination requirements of both Al and Ca. The coordination of Al is known to depend on neighboring cations, albeit Al is believed to be prominently four-fold coordinated^1^,^2^,^3^,^19^, as flagged by the most frequently observed value for the polyhedron O-Al-O bond angle (approximately equal to 109° 18,23). It has been suggested that the tendency of other network-modifying cations to charge-balance [AlO_4_]^5−^ polyhedra decreases with increasing ionic field strength, according to the sequence K^+^ >Na^+^ >Ca^2+^ >Mg^2+^ 19. Interestingly, the mobility of the Ca species varies in correlation to the shifting of Ca ions, from acting as network modifiers to being charge compensators of [AlO_4_]^5−^ tetrahedra, this trend increasing with decreasing CaO/Al_2_O_3_ mole ratio^9^.

The consistency of the SRO characteristics present in the amorphous slag has been highlighted in our precursor work, by Reverse Monte Carlo (RMC) fitting of experimental structure factors attained from high-energy X ray and neutron diffraction^1^,^2^,^3^. Here we utilize the SRO established in that work as a starting structure for MD calculations aimed at extracting the transport properties of the slag in the high temperature regime relevant to EAF operation. MD simulations are based on a Buckingham-type potential which has been optimized for an extensive range of slags of industrial interest, and which was specifically extended in the current work to account for interactions from the Cu and Cr species. This inclusion allows for a total of eight elements in the system (i.e. Fe, Si, Al, Mg, Ca, Cu, Cr and O); this renders the current effort as one of the most pragmatic MD studies of industrial slags from first principles, as the usual MD practice is to approach slags of up to four elements. A comprehensive array of SRO pair correlation statistics and physical properties of the melt are determined, principally focusing on the very important scales of electrical conductivity and viscosity.

## Methods

### Materials and chemical analysis

A quantity of the ferro-aluminosilicate slag was obtained from the industrial smelting plant of LARCO S.A., in Larymna, Greece. The material was collected off the EAF slag outlet at approximately 1673 K and was allowed to solidify at room temperature. In order to produce a pulverized material, the samples underwent further processing as described in more detailed elsewhere^2^. Chemical analyses of 0.1 g of the slag were carried out by HCl digestion and the constituent elements were determined via a Perkin Elmer 2100 atomic absorption spectrophotometer in solution. The elemental concentration of the slag in the current work is given in Table 1 (marked as Case 2).

### Computations

Classical MD based on system-optimized potentials offers an acceptable tradeoff between the ability to simulate mixed oxide supercells of the order of thousands of atoms and the requirement for prognostic accuracy of the atomic structure. This is particularly true for thermodynamics and transport properties in the high temperature regime^4^,^10^,^14^,^16^,^20^. The core of the MD calculation consists of solving the Newton equations of motion, associated with an assembly of particles (e.g. ions) interacting via a force field, the latter having been determined either empirically or from first principles. In the case of empirical force fields explicit to crystalline and molten silicates, each atom is mapped onto an effective point charge representing that atom within the oxide (e.g. for SiO_2_: q_Si_ = +4e and q_O_ = −2e). The exchange repulsion energy between the atoms is usually accounted for by a Born–Huggins–Mayer exponential term supplemented by dispersion forces; the latter are taken to be proportional to *C*/*r*^6^, where *C* is a constant and r is the interatomic distance between the atoms^10^.

Τhe interatomic interactions in the system of the current work were expressed by a Buckingham-type ionic force field. It is noted that the Buckingham potential may be derived from a Born-Mayer-Huggins (BMH) potential^4^,^9^,^14^ if in the latter expression the 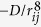 term is set to zero^21^. The Buckingham potential, 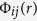, comprises an electrostatic interaction (i.e. Coulomb’s law for pairs of ions), a short-range repulsion interaction and attractive van der Walls forces using an 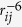 dependence determined by London, as shown below


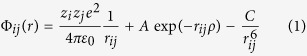


where e is the electron charge, 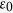 is the dielectric constant, *z*_*i*_, *z*_*j*_ are atom effective charges, *r*_ij_ denotes the interatomic distance between the i^th^ and j^th^ atoms. The quantities *A*, * ρ* and *C* are constants which are meant to be modified, such that the structure in the atomic supercell satisfies experimental datasets (e.g. the structure factor and/or the pair distribution function arising from total scattering of X ray or neutron sources)^1^,^2^,^3^. Here, parameters *A*, *ρ* and *C* for Cr and Cu were determined via force field optimization, as no relevant data exist in the literature (see section ‘Force field optimization’ below). The potential was additionally optimized for Fe-O interactions in order to obtain the best possible fit of pair correlation statistics between MD and RMC modelling of total scattering experiments^1^,^2^,^3^. The potential parameters for the Si, Al, Mg, Ca and O species were obtained directly from the work of Guillot and Sator^10^), as these were proven to be transferable across the entire range of industrial oxides.

### Pair correlation functions

The slag SRO was described via the Pair Distribution Function (PDF), symbolized as g(r) and expressed as


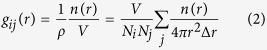


where *g*_*ij*_ denotes the partial PDF of the i^th^ and j^th^ atom species, N_i_ and N_j_ are the numbers of the species i and j, *V* is the volume of the system, and n(r) denotes the average number of the ions j surrounding ion i in a spherical shell defined by radii r ± Δr/2 4. The atomic coordination number (CN), expressed as *N*_*ij*_, was also derived on the basis of the PDF, as


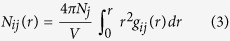


where *N*_*ij*_ corresponds to the i-j partial. SRO analysis of the atomic supercells was conducted by ISAACS^22^ and PRDF^23^.

### Force field optimization

The optimal values for parameters *A*, *ρ* and *C* (see Eq. 1) were determined by fitting the location of the first PDF peak at both room temperature and temperature above the melting point; the fitting was based on experimental PDF datasets which were determined by RMC modelling of high energy X-ray and neutron diffraction datasets of slag samples, acquired in our precursor work^1^,^2^,^3^. The optimization simulations were carried out in the canonical (NVT) and isothermal-isobaric (NPT) ensemble. Force field optimization involved state points at a pressure of 1 atm and at two different temperatures. The Pressure-Volume-Temperature (PVT) state point at 298.15 K (the low temperature bound) corresponded to room temperature while the state point at the high temperature of 1773.15 K referred to the material’s liquid state. The optimization procedure consisted of minimization over the potential parameters *A*, *ρ* and *C* of the following dimensionless quantity^24^.


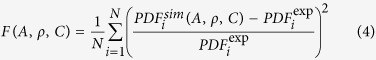


where *N* is the number of PVT state points, 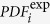 is the experimental and 
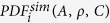
 is the interatomic distance of the first PDF peak at state i for the parameter set (*A*, *ρ*, *C*). Minimization of function *F* was performed by the Simplex linear method as the latter is guaranteed to locate the global minimum. Since *F* has no analytical derivative, use of derivative-based minimization methods would be computationally expensive, requiring three simulations per state point and iteration for the calculation of both the value and the derivative of the function. In contrast, the Simplex method requires n + 1 simulations and one additional simulation per state point and iteration, where n is the number of the parameters to be optimized^24^. At each state point (i.e. for each set of parameters *A*, *ρ* and *C*) a 120 ps MD simulation was performed in the NVT ensemble, in order to determine the location of the first PDF peak. During post MD-processing, the first 20 ps of the trajectory of each simulation were discarded. In this set of simulations, the values of the parameters *A*, *ρ* and *C* were taken from the work of Guillot and Sator^10^; in the case of Cr and Cu, the initial parameter values were approximated by setting them equal to the original parameters^10^. for Fe and Mg, respectively. The parameters of the optimized potential are presented in Table 2.

To ensure the transferability of the interaction potential with melt composition, the effective charges assigned to each of the atom species were kept fixed for all compositions studied^10^,^20^. The requirement for transferability of the potential and taking into account the presence of Cr and Cu, imposes that the following holds true^10^.


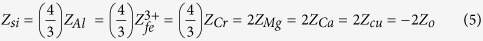


where *Z* is the charge of the oxides.

### MD simulations

All simulations were performed with GROMACS^31^,^32^,^35^,^36^. The equations of motion were integrated with the leap-frog algorithm (a time step of 1 fs was used^24^) in a cubic simulation cell to which periodic boundary conditions were imposed^9^. The side lengths of the simulation cell were dependent on density and temperature. Long range electrostatic corrections were taken into account via use of the Particle Mesh method, while standard van der Waals long range corrections for potential energy and pressure were applied^4^,^9^,^25^,^26^. The Nosé–Hoover thermostat^26^ was applied in all runs. At each temperature, energy minimization was achieved by a quasi-Newtonian algorithm based on the low-memory Broyden-Fletcher-Goldfarb-Shanno (l-bfgs) approach^27^,^28^. Calculation of transport properties involved sampling from both the NPT and NVT ensembles at each temperature step. The length of each production run was 16 ns (16000000 time steps). To evaluate the influence of the size of the system on the properties of the melt, we performed several tests for supercell sizes ranging from 3003 to 162000 atoms. Sensitivity analysis of thermodynamic properties did not reveal significant effects in respect to supercell size, apart from the anticipated reduction of statistical noise^10^. Consequently, we chose a supercell which consisted of 6002 atoms, comprising a good compromise between accuracy and computational cost.

### Determination of physical properties

The properties of the slag determined by post-MD analysis were the density, melting point, heat capacity at constant pressure (C_P_) and at constant volume (C_V_), the thermal expansion coefficients obtained using the NPT ensemble as well as the diffusivity, electrical conductivity and viscosity using the NVT ensemble. These are discussed below.

#### Density

The density *ρ* at constant pressure follows from the mass, *M*, of the system divided by its volume *V*^26^


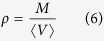


where the bracket denotes time averaging over the simulation period.

#### Viscosity

The shear viscosity was calculated from the fluctuations of the off-diagonal elements of the pressure tensor using the Green-Kubo formula reformulated into the Einstein relation^29^.


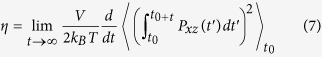


where *V* is the volume of the simulation box, k_*B*_ is the Boltzmann constant, *T* is the temperature, *P* is the pressure and *t*_0_ is the initial time step. Also, the system’s shear viscosity was evaluated via the Stokes-Einstein formula^6^,^29^,^30^.


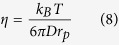


where *r*_*p*_ is the radius of the particle and *D* is the coefficient of self-diffusion. Additionally, the shear viscosity was determined from transverse-current correlation functions for plane waves^31^. The shear viscosity obtained using this method is dependent on the wavenumber k = ||**k**||. To obtain the macroscopic shear viscosity one needs to extrapolate to k = 0. Palmer^31^ argues that since the viscosity in one dimension should be a symmetric function of k, one can approximate the viscosity to a third order by^32^.


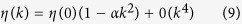


#### Diffusivity

For each atomic constituent, the MSD  -  which indicates the average displacement of a tagged atom during a fixed time t, calculated by summing the square of distance over all the atoms and dividing by the number of atoms, *N* - is calculated as follows


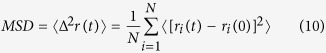


where *r*_*i*_(*t*) is the position of atom *i* at time *t*. The angular brackets indicate an average over the positions of the atoms at time *t* = 0 5,6. The coefficient of self-diffusion of a particle may be obtained from the MSD for sufficiently long simulation times (over 10 ns) by use of the Einstein equation^8^,^9^,^24^.


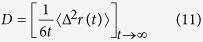


#### Electrical conductivity

Within the framework of linear response theory (Kubo 1996)^33^, the electrical conductivity of an ionic liquid is given by^10^.


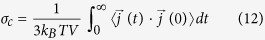


where the charge current 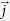 is defined by


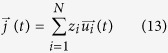


for which *V* the volume of the sample, *z*_*i*_ the charge carried by ion i, 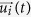 is the ion velocity and *N* the total number of ions. Alternatively, Eq. 13 can be rewritten in a more convenient form in terms of the mean square displacement of the ions


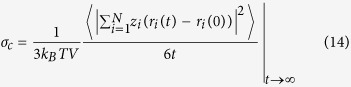


The ionic conductivity of the ferro-aluminosilicate slag was calculated via the Nernst–Einstein equation^6^. This relation assumes that each ion is mobile independently from the rest. Consequently, Eq. 14 reduces to,


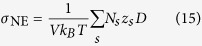


where σ_NE_ is the ionic conductivity, *N*_*s*_ is the number of ions in the simulation cell, *z*_*s*_ is the electron charge^10^,^11^. In the current work, the electrical conductivity was calculated via Eq. 15; however, for the sake of completeness, we point out that the exact expression of the electrical conductivity is of the form


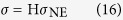


where H, the Haven ratio, i.e. the ratio of the tracer diffusion coefficient to the charge diffusion coefficient^34^. In molten salts H < 1 because the anion-cation interactions tend to decrease the conductivity^10^.

#### Coefficient of thermal expansion

The coefficient of thermal expansion, α, was estimated from fluctuations in the volume of the system using the relation.


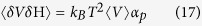


where *α*_*p*_ is the volumetric thermal expansion coefficient, H is the enthalpy, and δ indicates the fluctuations, for which^26^


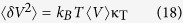


where *κ*_T_ is the isothermal compressibility.

#### Specific heat

The specific heat at constant pressure *Cp* was estimated using the relation^26^.


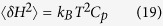


while the specific heat at constant volume *C*_*v*_ was estimated by the expression


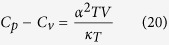


where *V* is the volume of one kilogram of the substance when *C*_*p*_ and *C*_*v*_ are also expressed per kilogram and *κ*_T_ is the isothermal compressibility^26^,^29^.

## Results and Discussion

### SRO

**Figure 1:**
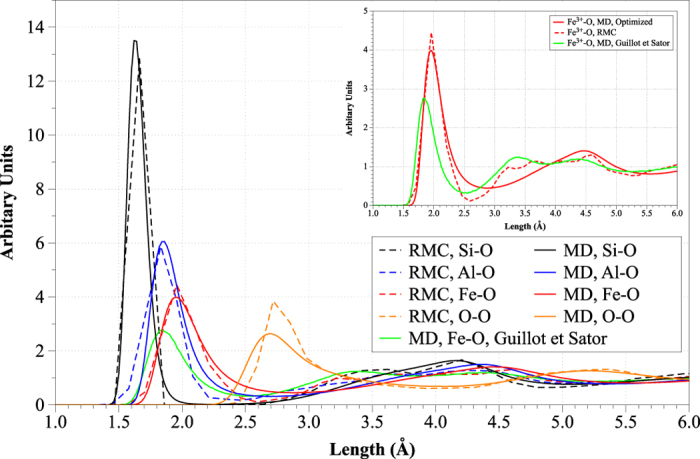
RDFs calculated by RMC (dotted curves) and MD at 1773 K. The total CN corresponding to the Si-O, Al-O and Fe-O partials for MD and RMC simulations are 4.00, 3.73, 3.71 and 3.97, 3.87 and 3.08 respectively.

The optimal values of the parameters *Α*, *ρ* and *C* (see Eq. 1) as determined here for Fe, Cr and Cu are listed in Table 2, along with the original values of the parameters for the rest of the interactions^10^. A comparison between the PDFs at 1773.15 K calculated in the current work and those obtained from the RMC fitting of our experimental data^1^,^2^,^3^ is portrayed in Fig. 1. Also, the inset of Fig. 1 displays the Fe-O PDF obtained by MD simulations using the optimized potential parameters, compared with RMC as well as with the Fe-O resulting from the analysis by Guillot and Sator. As seen, the MD-predicted Si-O bond length was fractionally underestimated (by approximately 0.04 Å) in respect to RMC. As this difference was considered negligible, no modifications in the parameters of the potential were made for Si. Similarly fractional were the deviations between MD and RMC for Al, Fe and O bond lengths with the oxygen species (all deviations were of the order of 0.01 Å – see Fig. 1). The PDF curves arising from MD were smoother than those by RMC; this is owing to the use of a considerably large array of sampled MD conformations which lead to better sampling compared to inverse modelling. Interestingly, the average coordination numbers (within the first coordination shell) of Si-O, Al-O and Fe-O calculated by MD were in accordance to RMC estimations (see the values shown in the caption of Fig. 1).

Nearest-neighbor distances for the Si–O, Al–O, Fe-O and O-O partials were equal to 1.62, 1.86, 1.96 and 2.70 Å respectively, in perfect agreement with the literature^1^,^2^,^3^,^16^,^35^,^36^,^37^,^38^,^39^,^40^,^41^. These distances for the Fe-Fe and Si-Si partials were 3.38 and 3.12 Å respectively, again, comparing favorably against literature values^1^,^2^,^3^. From the data, the cutoff radii of the most important oxygen partials at 1773.15 K were also determined, as the upper limit (interatomic distance) of the first PDF peak. These radii were 2.24, 2.42, 2.72 and 3.82 Å for Si-O, Al-O, Fe-O and O-O respectively; apart from the O-O partial, all previous interatomic distances lie within the first coordination cell.

Table 3 presents the most important bonding features within the first coordination shell and at temperature steps of 200 K across the temperature range examined. Network-forming cations such as Si and Al were principally in four-fold coordination with respect to oxygen ligands. In the case of Si, the shift to higher temperatures caused an increase of the percentage of penta-coordinated centers at the expense of 4-fold coordination. Overall, the average coordination number of Si consistently remained equal to four, in tight agreement with our previous experimental work on X ray diffraction of levitated melt slags^1^,^2^,^3^. In the case of Al and Fe, average coordination numbers ranged between 3.7 and 4.6 for the former and from 4.0 to 4.9 for the latter (the lower CN detected at higher temperatures). A side-to-side comparison of coordination features between the two temperature extrema (1273.15 and 2273.15 K) revealed that 2-, 3- and 4-fold coordination increased with increasing temperature at the expense of 5- and 6-fold coordination. This coordination trend in respect to increasing temperature is in agreement with other observations made in the literature, suggestive of an increasingly depolymerized network^9^,^48^.

**Figure 2:**
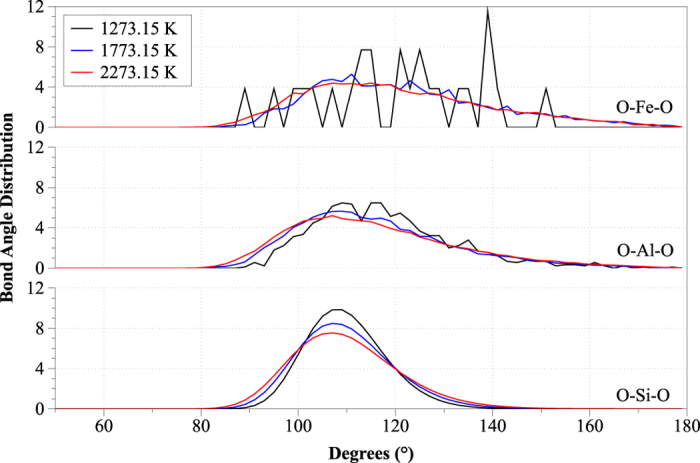
Distributions of the (**a**) O-Si-O (b) O-Al-O (c) O-Fe-O angles for temperatures of 1273.15, 1773.15 and 2273.15 K.

Within the first coordination shell, the bond angle distributions (BAD) for O-Si-O, O-Al-O and O-Fe-O were also calculated using 1600 frames (time step of 100 ps over the 16 ns simulation time) at each state point as shown in Fig. 2. The O-Si-O BAD peaks for 1273.15, 1773.15 and 2273.15 K were located at 107.0-109.0, 107.0 and 107.0 degrees, respectively (see Fig. 2). This indicates a gradual deformation of Si tetrahedra with increasing temperature, while at the low temperature the tetrahedra were almost canonical, as signified by the proximity of the O-Si-O BAD peak (located at 109 degrees) to the ideal tetrahedral angle of 109.5 degrees. In the case of the O-Si-O BAD, a shift towards lower angles at high temperatures was observed, owing to the combined effect of a decrease of the number of SiO_4_ units (see Table 3) and of an increase of the penta-coordinated Si (leading to a shift of the curve towards lower angles)^42^. In the case of O-Al-O, the BAD was broader and its peak was lower than that of the O-Si-O BAD, indicating substantial irregularity in the shape of Al-centered polyhedra^19^. For temperatures of 1273.15, 1773.15 and 2273.15 K the primary O-Al-O peaks were located at 109.0, 107.0 and 107.0 degrees respectively, in agreement with literature values^1^,^2^,^3^,^43^. Al was 4-fold coordinated, its CN ranging between 3.8 and 4.6. On the basis of experimental observations, at higher temperatures peak broadening is due to the increase of 2- and 3-fold coordinated Al ions^44^. In the case of the O-Fe-O BAD at 1273.15 K, peaks were located at 115.0, 121.0, 125 and 139 degrees. The discontinuous shape of the O-Fe-O BAD at 1273.15 K suggests that there are no preferred orientations of the O-Fe-O plane in contrast to O-Si-O, in which the regular shape of the distribution suggests the presence of SiO_4_ tetrahedra^45^. At higher temperatures (1773.15 and 2273.15 K), the BAD peaks were shifted to 109.0 degrees.

The principal coordination features of the oxygen species in the first coordination shell are presented in Table 4. As a general observation, MD simulations lead to higher fractions of Fe-O and Si-O bonds in comparison to the results obtained from our RMC simulations^1^,^2^,^3^. The most substantial difference between the results from MD and RMC was related to the fraction of uncoordinated O atoms; perhaps counter-intuitively, there were no uncoordinated O in the MD supercell, irrespective of temperature. On the contrary, the number of NBOs increased with increasing temperature from 1273.15 to 2273.15 K. More specifically, upon rising temperature, NBOs linked to Si, Fe, Mg and Al increased by 9.12, 2.38, 0.35 and 0.49% respectively. This feature suggested that an increase in temperature tends to cause a dismantling of rings structures, as also indicated by BADs. Interestingly, MD re-affirmed a previous observation, that the majority of NBOs were preferentially connected to Si than Al tetrahedra^4^.

### Ring statistics

**Figure 3:**
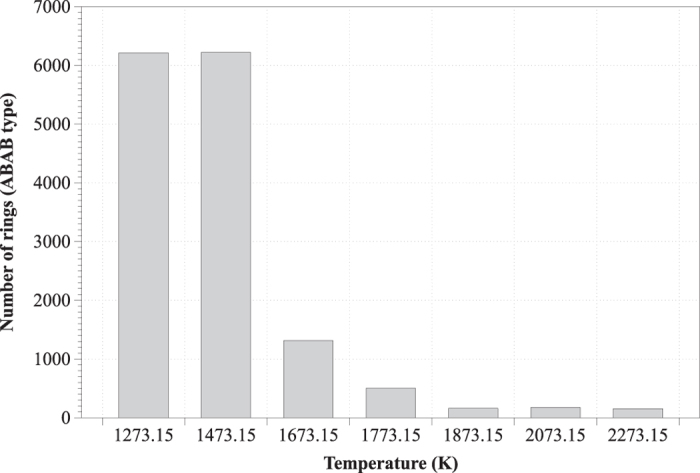
Ring size distribution in respect to the temperature.

In silicate systems, rings are defined as loops of T-O links (T = Si, Fe, Al) and the ring size distribution is a measure of medium range order^16^. The distribution of ring sizes of the ABAB type (e.g. Si-O-Si-O)^22^ is presented in Fig. 3. Overall, ring sizes appeared to vary between the values of 3 and 30. At temperatures above the melting point, the largest rings had sizes from 6 to 10, while at lower temperatures sizes ranged between 24 and 30. Temperature transition from 1473.15 to 1673.15 K revealed a pronounced ring size gap, indicating a shift of the slag from the solid to the liquid state. At the high temperature extremum (2273.15), the ABAB-type rings were altogether dismantled (only 155 rings were observed, in contrast to the 6211 rings detected at the lowest temperature). Indicatively, at 1773.15 and 2273.15 K the number of any type of rings was 3500 and 2307, respectively.

**Figure 4:**
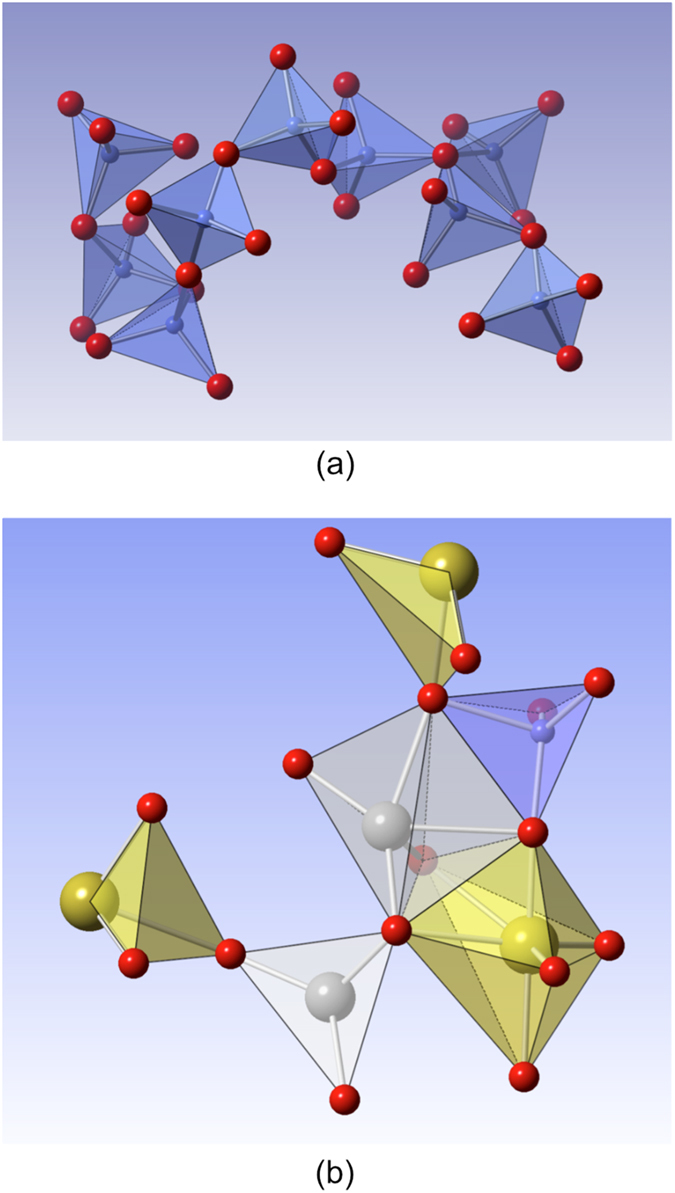
(**a**) Silicate chain as obtained at 1773.15 K. (**b**) Ferro-aluminosilicate chain obtained at 1773.15 K describing the face, edge and corner sharing tetrahedra. The color coding is as follows: Yellow – Fe, Blue – Si, Gray – Mg and Red – Oxygen.

On the basis of the SRO data and of the ring statistics established, a typical chain composed of interconnected Si tetrahedra is reconstructed in Fig. 4a. Also, a cartoon of the most probable interconnects found in the system is shown in Fig. 4b.

### Density, melting point, thermal expansion coefficient and heat capacity

**Figure 5:**
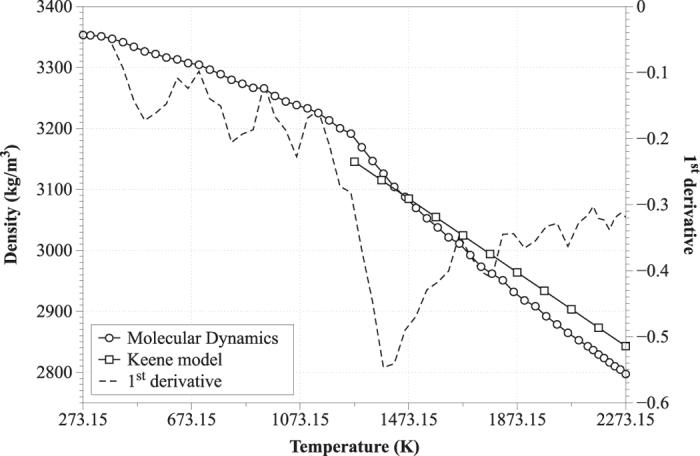
Variation of the density with the using MD simulations and the empirical model of Keene.

The distribution of mass density is shown in Fig. 5. At room temperature (298.15 K) the density obtained by MD was 3353.00 ± 14 kg/m^3^, in excellent agreement with experimental data^1^,^2^,^3^. In the temperature range 1473.15–1773.15 K (which is characteristic of the EAF operation) the density varied between 3083.19 ± 11 and 2965.97 ± 12 kg/m^3^; the lower density value is in good agreement with our measurement of 2940 kg/m^3^ based on levitation experiments^2^. In the same temperature range, the density range calculated by MD is also similar to the range of 3084.76–2994.03 kg/m^3^ estimated via the empirical model of Keene, a reference model for an array of mixed oxide melts owing to its prognostic accuracy^5^,^6^. From the first derivative of the distribution of mass density in respect to temperature, the melting point of the system was determined to be equal to 1380 K. This value is acceptable, based on our own industrial measurements at the EAF slag outlet which indicated melt temperatures of 1570–1780 K.

**Figure 6:**
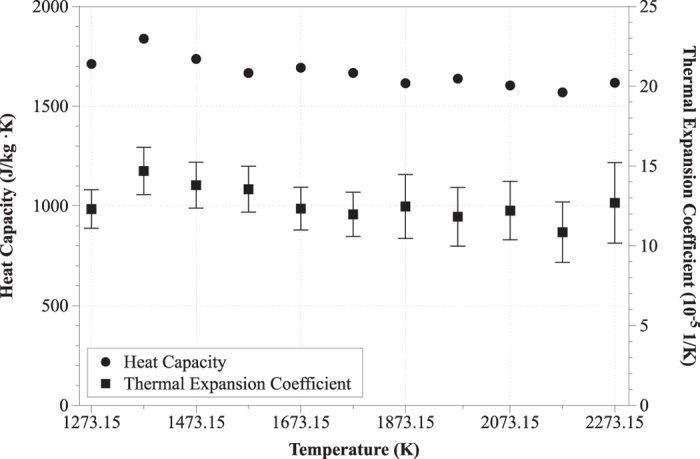
Heat capacity (J/(kg·K)) and thermal expansion coefficient (1/K) in respect to temperature.

The variation of heat capacity and of the thermal expansion coefficient in respect to temperature is portrayed in Fig. 6. The standard deviation of heat capacity is below 2% and consequently may not be displayed in Fig. 6. The thermal expansion coefficient was found to lie between (10.85 ± 1.9)×10^−5^ and (14.69 ± 1.5)×10^−5^ 1/K with an average value of 12.60·10^−5^ 1/K in agreement with data by Mills^5^. Similarly, the heat capacity varied between 1569.21 ± 23 and 1837.97 ± 23 J/(kg·K) with an average value of 1668.58 J/(kg·K). It was also observed that heat capacity decreased with increasing temperature which was interpreted as the progressive loss of liquid shear resistance^46^,^47^.

### Self-Diffusion, electrical conductivity and viscosity

**Figure 7:**
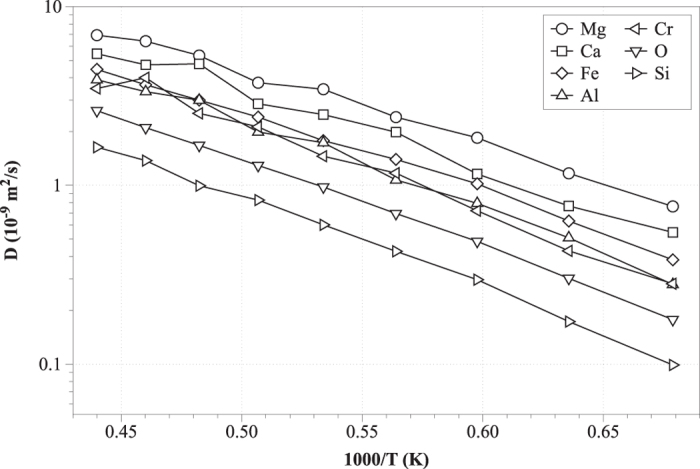
Self-diffusion coefficients of Fe, Si, Al, Mg, Ca, Cr and O as a function of temperature.

In order obtain accurate statistics in the calculations related to self-diffusion and viscosity, 16 ns simulations were performed. Fig. 7 illustrates the self-diffusion coefficients as a function of reciprocal temperature. Due to the fact that the standard deviation of the values was below 1%, it is not presented in the latter figure. Mg and Ca proved to be the most diffusive species, as they are both network modifying and charge balancing cations^4^,^5^,^9^. The least diffusive species were O and Si. Amphoteric ions (Fe, Al and Cr) exhibited increased diffusivity when these acted as network modifiers and low diffusivity when they took on network forming roles. On the basis of the data, we established the diffusivity tendency to be D_Mg_ > D_Ca_ > D_Fe_ > D_Al_ > D_Cr_ > D_O_ > D_Si_, where D is the coefficient of self-diffusion. Similar diffusivity behavior has also been reported by Zheng *et al.* who reported a sequence of the sort D_Ca_ > D_Al_ > D_O_ > D_Si_^9^. The main reason underpinning the low diffusivity of the Si and O atoms, is the creation of silicate chains within the slag. Also, the diffusivity of O depends on the balance between the concentrations of Si and Al. Due to the low diffusivities of O, Si and Al it is plausible to assume that O atoms are in motion in conjunction with the centres of Si- and Al tetrahedra, as also suggested elsewhere^8^. Also our analysis indicated that increasing fraction of network forming oxides in the system (e.g. based on polyhedra involving Si, Al centers) mediates melt polymerization. We consider that network forming oxides reduce the coefficients of self-diffusion and cause the melt to become more silicic; the net effect of this transition is a reduction of the electrical conductivity and an increase of viscosity^10^.

**Figure 8:**
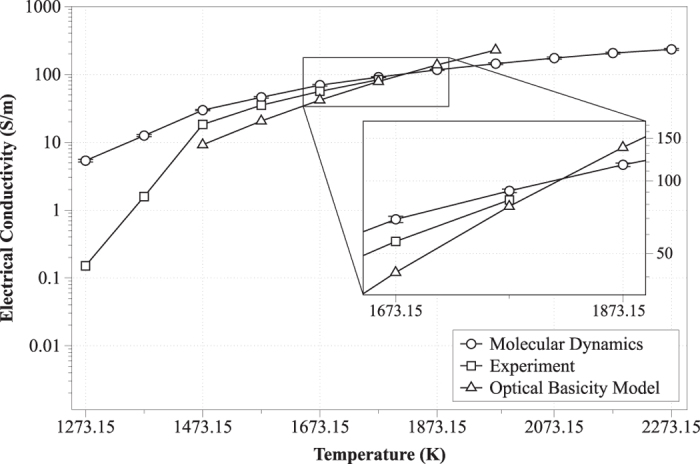
Electrical conductivity in respect to temperature.

The electrical conductivity of the slag was calculated via Eq. (15), and the results are presented in Fig. 8; the computed values were compared with experimental data acquired on a sample of very similar stoichiometry, as well as with predictions by the Optical Basicity empirical model^5^,^6^. From the data it is apparent that the Nernst-Einstein equation overestimates the electrical conductivity for temperatures lower than the melting point (see MD vs. experiment trends in Fig. 8). This behavior is most probably owing to the fact that, at low temperatures, the slag is a semiconductor. Temperature increase favors an increase in electrical conductivity, most likely because the slag becomes more depolymerized^10^. The range of the electrical conductivity values obtained here from both MD and experiment compared favorably against values from a comprehensive array of related systems (10–150 S/m)^20^. The tight dependency between conductivity and NBO/O suggests that the diffusion of the NBOs is more efficient^48^. Consequently, slag depolymerization related to motion of NBOs explains the increase of the electrical conductivity. In our case the increase of NBOs in the range 1473.15–2273.15 K was 646% (*i.e.* the increase from 1.83% to 13.67%). Also, the material’s diffusivity exhibited a linear behavior in respect to temperature D_1273.15_ = (4.06 ± 0.0142) × 10^−11^ m^2^/s and D_2273.15_ = (3.1845 ± 0.0641)×10^−9^ m^2^/s.

**Figure 9:**
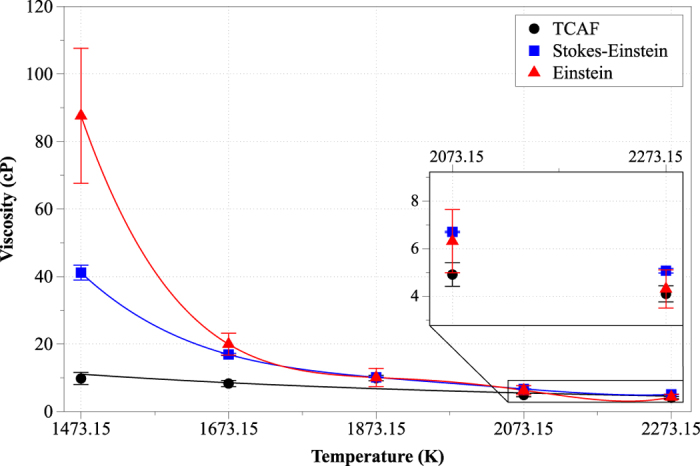
The dependence of slag viscosity on temperature.

Calculation of the shear viscosity of the slag involved several runs using supercells from 6000 to 162000 atoms and Coulomb cutoffs in the range 1.2–2.2 nm. At 2173.15 K the viscosity determined by the TCAF model^32^. ranged between 7.79 and 8.23 cP^6^. Due to the negligible variations of viscosity in respect to the number of atoms in the supercell, a final combination of 6000 atoms with a 2.0 nm Coulomb cutoff was used for all consecutive simulations. At temperatures below 1473.15 K, the viscosity values were extremely high (e.g. 1359 ± 366 cP at 1273 K), which suggested that the simulation reached the solid state. As mentioned previously, the viscosity is correlated to the degree of melt polymerization; at high temperatures the viscosity is low due to the increase of NBOs. At high temperatures, where the slag acts as Newtonian fluid^7^,^12^,^49^,^50^, all three models yielded very similar results (i.e. Einstein 4.3 ± 0.8, Stokes-Einstein 5.06 ± 0.09 and TCAF 4.09 ± 0.34 cP). At temperatures close to the system liquidus (i.e. 1380 K) the melt does not behave as a Newtonian fluid and therefore only the Stokes-Einstein and the Einstein methods were of the same order of magnitude (i.e. Einstein 87.64 ± 20, Stokes-Einstein 32.78 ± 2 and TCAF 9.80 ± 1.8 cP). A comparison of viscosity results as a function of temperature for each of the methods used is shown in Fig. 9. The viscosity values obtained at 1673.15 and 1873.15 K were in the range 13.48 ± 0.3–19.97 ± 3.25 cP for the former and 9.9 ± 0.8–10.14 ± 0.1 cP for the latter temperature, while at 2273.15 K the viscosity range was 4.09 ± 0.34 –5.06 ± 0.1 cP, in agreement with the literature^51^. To portray the consistency of our methodology, we calculated the viscosity of the melt via the empirical models of Lida, Forsbacka Mills^5^. We found that these models exhibit pronounced deviations in their predictions; for example, at 1773 Κ the respective viscosity values by the Lida, Forsbacka and Mills models were 0.04, 6.47 και 0.005 cP respectively, indicating a difference of tree orders of magnitude. Only the Forsbacka model was found to agree with our MD-based results in the temperature range 1673.15–1773.15 Κ.

### Transferability of the interatomic potential

The transferability of the optimized Buckingham-type potential is warranted solely within the premise of mixed oxides of industrial usage (see elemental concentration limits under ‘Industrial slags’ in Table 1). Primarily, the original force field parameters for Si, Al, Mg, Ca and O as established in the work by Guillot and Sator^10^ are transferable.

As shown in Table 1, the current work has tested the integrity of the force field on four different slag concentrations for a range of temperatures (see Cases 1 to 4 in Table 1). As laid out previously, the location of the first PDF peak for Cases 1 to 4 was consistently in agreement to RMC, although, as expected, there were mild differences in atomic coordination since the stoichiometries of Cases 2 to 4 were slightly different to that of the RMC supercell. In particular to Case 1, the density predicted was equal to 2956.00 ± 16 kg/m^3^ in full accordance to the experimentally established value of 2940.00 kg/m^3 ^1,2,3; We also calculated the system density based on the parameters by Guillot and Sator and found it to be 3010.95±11.7 kg/m^3^, which is considerably less accurate than the prediction by the optimized force field.

To further assess the transferability of the optimized potential, electrical conductivity calculations were performed for Cases 2 to 4, containing the lowest and highest Cr concentrations in the slag. As seen from the data, the electrical conductivity for Cases 3 and 4 was 90.87 ± 1.3 and 91.93 ± 1.5 S/m, respectively. This indicates a less than 0.05% difference between the two values and an average difference of 0.57% from the value of 90.92 ± 1.5 S/m, corresponding to the original slag composition of Case 2. As these deviations are negligible, we deem that, to the extent that Cr and Cu concentrations remain within the range of industrial interest, the optimized potential is transferable.

## Conclusions

A Buckingham-type interatomic MD potential was extended for the presence of Cr and Cu species in a high temperature slag of industrial importance. On the basis of 16 ns production runs, there was consistency between the calculated PDF values and data attained from the inverse modelling of our total scattering experiments. Additionally, the optimized potential produced SRO and ring statistics which were in full accordance with the literature. The material’s calculated density was 3087.60–2961.50 kg/m^3^ within the temperature range 1473.15–1773.15 K. The lower density, in particular, was in excellent agreement with a measured value of 2940 kg/m^3^ obtained from our levitation experiments. Upon this consensus, we proceeded to calculate transport properties with emphasis on electrical conductivity and viscosity. It was determined that the conductivity calculated for three separate slag stoichiometries compared most favorably against experimental data. Moreover, the MD model predicted a liquidus of 1380 K which was in accordance with experimentally established temperatures at the EAF slag outlet. Based on SRO, the model consistently made proper predictions of electrical conductivity, tightly correlated to our experimental results. The three independent calculations of 90.87, 90.92 and 91.93 S/m confirmed that, within the limits of engineering relevance, the MD model is consistently accurate. Moreover, the predicted Cp values (1569.21–1837.97 J/(kg·K)) were in full accordance with the literature. Based on the integrity of our results, the optimized potential is transferable.

## Additional Information

**How to cite this article**: Karalis, K. T. *et al.* Bona-fide method for the determination of short range order and transport properties in a ferro-aluminosilicate slag. *Sci. Rep.* **6**, 30216; doi: 10.1038/srep30216 (2016).

## Figures and Tables

**Table 1:** Elemental concentration of the slags studied in the current study and concentration limits for slags of industrial interest (designated as ‘Industrial slags’).

Element	Industrial slags	Concentration (w.t.%)			
		Case 1	Case 2	Case 3	Case 4
Fe	24.47–32.51	26.52	29.41	29.92	29.93
Si	18.11–19.44	19.16	18.42	18.73	18.74
Al	3.16–3.92	3.38	3.17	3.22	3.22
Mg	3.85–5.07	4.26	3.86	3.93	3.93
Ca	2.60–4.23	2.59	2.63	2.68	2.68
Cr	1.60–2.60	2.65	1.69	1.60	2.60
Cu	0.02–0.04	0.10	0.02	0.02	0.02
O	35.55–41.70	41.34	40.81	39.88	38.88
	Temperature low	298	298	—	—
	″″ high	1773	2273	1773	1773
	″″ step	—	5	—	—
	Conductivity (S/m)		90.92	90.87	91.93

The slag used for force field optimization is designated as Case 1. The slag marked as Case 2 was used for the determination of SRO and transport properties. Cases 3 and 4 were used in the assessment of force field transferability (Cases 3 and 4 included 44 and 71 Cr atoms respectively, while both systems included a single Cu atom). For each of Cases 1 to 4, the temperatures used in the MD simulations are also shown.

**Table 2:** Numerical values of the parameters Α, ρ and C (see Eq. 1) as these were established via PDF fitting of experimental total scattering data^1^,^2^,^15^.

Name	Charge z(e)	A (kJ/mole)	ρ (nm^−1^)	C (kJ nm^6^/mole)
Fe^3+^	1.4175^10^	1178340.00	52.631579	0.000000^10^
Cr	1.4175	2753544.30	56.139535	0.003336
Al	1.4175^10^	2753544.30^10^	56.139535	0.003336^10^
Ca	0.9450^10^	15019679.10^10^	56.179775	0.004077^10^
Mg	0.9450^10^	3150507.40^10^	56.179775	0.002632^10^
Si	1.8900^10^	4853815.50^10^	62.111801	0.004467^10^
Cu	0.9450	3150507.40	59.179775	0.001853
O	−0.9450^10^	870570.00^10^	37.735849	0.008210

It is noted that these parameters correspond to cation-oxygen and oxygen-oxygen interactions. Cation-cation interactions are accounted for only by coulombic repulsive forces. Parameters taken from the work by Guillot and Sator are marked by the corresponding reference.

**Table 3:** Principal coordination features (CN) within the first coordination shell.

	CN	Temperature (K)						
		1273.15	1473.15	1673.15	1773.15	1873.15	2073.15	2273.15
		% of feature						
Si-O	3	0.10	—	0.10	0.30	0.30	—	0.52
	4	99.58	99.06	98.74	98.74	97.70	96.97	97.70
	5	0.20	0.80	1.14	0.80	1.67	2.40	1.67
	2	—	—	—	—	—	1.74	2.32
	3	—	8.72	7.55	4.65	9.88	16.27	27.90
Al-O	4	43.02	70.34	65.11	65.11	57.55	68.02	58.72
	5	49.41	18.60	23.83	16.16	27.90	12.79	9.88
	6	7.55	1.74	3.48	2.90	2.90	0.58	0.58
	1	—	—	—	—	—	—	1.95
	2	1.69	2.21	2.73	4.55	5.591	6.63	19.63
Fe-O	3	15.99	21.84	27.82	30.68	32.25	34.33	48.24
	4	56.17	53.44	54.74	51.23	49.28	46.16	28.21
	5	23.53	21.06	14.43	12.35	11.83	11.44	1.82
	6	2.60	1.43	—	0.52	—	—	—

The feature consists of a central atom (shown as the first species in the pair, e.g. Si in the pair Si-O) coordinated by atoms of the second species (e.g. O in the pair Si-O). The percentage of each feature is in respect to the total number of coordination features present in the supercell for that central atom.

**Table 4:** MD-generated oxygen coordination features within the first coordination shell of atomic structure.

Coordination Feature	Temperature (K)					
	1273.15	1473.15	1673.15	1873.15	2073.15	2273.15
1Fe, 1Si	22.18	25.51	22.99	23.77	22.75	23.2
1Si	1.50	1.63	3.56	3.62	4.53	**10.62**
2Si	19.39	17.84	19.56	19.18	17.19	20.36
1Fe	0.16	0.10	0.72	0.69	0.69	2.54
1Fe, 2Si	0.77	1.04	1.10	0.93	1.47	1.15
1Si, 1Mg	0.42	2.92	**4.26**	**4.02**	**4.48**	**5.36**
2Fe	**2.79**	**2.89**	**4.15**	**4.74**	**4.61**	**5.17**
1Si, 1Al	**5.55**	**5.52**	**5.90**	**5.31**	**5.74**	6.38
1Fe, 1Si, 1Mg	6.30	5.84	5.01	**4.08**	**3.91**	**2.73**
1Fe, 1Mg	0.53	0.45	0.67	0.96	0.96	**1.58**
1Mg	0.02	—	0.027	0.03	—	0.37
1Si, 2Fe	6.73	6.6	4.37	**3.38**	3.43	**2.17**
1Si, 1Fe, 1Al	3.38	**2.28**	**2.17**	0.99	**2.06**	**1.20**
1Fe, 1Al	0.96	1.44	**2.01**	**2.03**	**2.09**	**2.65**
—	—	0.03	—	—	—	0.13
2Si, 1Mg	**1.2**	**1.23**	**1.39**	1.02	**1.18**	0.80
2Si, 1Al	0.26	0.26	0.26	0.45	0.13	0.45
1Al	0.02	0.1	0.1	0.08	0.08	0.51
1Si, 1Mg, 1Al	2.06	**1.04**	**1.26**	**1.15**	—	**0.96**

The features highlighted in bold fall within ±30% of the respective values determined by RMC fitting of total scattering data carried out in our precursor work^1^^–^3. Each coordination feature is represented as a percentage of the total number of oxygen-related features in the supercell.
