# Erratum: Bona-fide method for the determination of short range order and transport properties in a ferro-aluminosilicate slag

**DOI:** 10.1038/srep33765

**Published:** 2016-09-27

**Authors:** Konstantinos T. Karalis, Dimitrios Dellis, Georgios S. E. Antipas, Anthimos Xenidis

Scientific Reports 6: Article number: 3021610.1038/srep30216; published online: 07 26 2016; updated: 09 27 2016

In this Article, the rows in Table 3 are misaligned. The correct Table 3 appears below

## Figures and Tables

**Table 3:** 

		Temperature (K)						
		1273.15	1473.15	1673.15	1773.15	1873.15	2073.15	2273.15
	CN	% of feature						
Si-O	3	0.10	—	0.10	0.30	0.30	—	0.52
	4	99.58	99.06	98.74	98.74	97.70	96.97	97.70
	5	0.20	0.80	1.14	0.80	1.67	2.40	1.67
Al-O	2	—	—	—	—	—	1.74	2.32
	3	—	8.72	7.55	4.65	9.88	16.27	27.90
	4	43.02	70.34	65.11	65.11	57.55	68.02	58.72
	5	49.41	18.60	23.83	16.16	27.90	12.79	9.88
	6	7.55	1.74	3.48	2.90	2.90	0.58	0.58
Fe-O	1	—	—	—	—	—	—	1.95
	2	1.69	2.21	2.73	4.55	5.591	6.63	19.63
	3	15.99	21.84	27.82	30.68	32.25	34.33	48.24
	4	56.17	53.44	54.74	51.23	49.28	46.16	28.21
	5	23.53	21.06	14.43	12.35	11.83	11.44	1.82
	6	2.60	1.43	—	0.52	—	—	—

